# Performance of ChatGPT on the Peruvian National Licensing Medical Examination: Cross-Sectional Study

**DOI:** 10.2196/48039

**Published:** 2023-09-28

**Authors:** Javier A Flores-Cohaila, Abigaíl García-Vicente, Sonia F Vizcarra-Jiménez, Janith P De la Cruz-Galán, Jesús D Gutiérrez-Arratia, Blanca Geraldine Quiroga Torres, Alvaro Taype-Rondan

**Affiliations:** 1 Academic Department USAMEDIC Lima Peru; 2 Facultad de Ciencias de la Salud, Carrera de Medicina Universidad Científica del Sur Lima Peru; 3 School of Medicine Universidad Nacional de Piura Piura Peru; 4 Comité Permanente Académico Sociedad Científica Médico Estudiantil Peruana Lima Peru; 5 Centro de Investigación de Estudiantes de Medicina Tacna Peru; 6 School of Medicine Universidad de San Martin de Porres - Filial Norte Chiclayo Peru; 7 Unidad de Investigación Para la Generación y Síntesis de Evidencias en Salud Vicerrectorado de Investigación Universidad San Ignacio de Loyola Lima Peru; 8 EviSalud - Evidencias en Salud Lima Peru

**Keywords:** medical education, generative pre-trained transformer, ChatGPT, licensing examination, assessment, Peru, Examen Nacional de Medicina, ENAM, learning model, artificial intelligence, AI, medical examination

## Abstract

**Background:**

ChatGPT has shown impressive performance in national medical licensing examinations, such as the United States Medical Licensing Examination (USMLE), even passing it with expert-level performance. However, there is a lack of research on its performance in low-income countries’ national licensing medical examinations. In Peru, where almost one out of three examinees fails the national licensing medical examination, ChatGPT has the potential to enhance medical education.

**Objective:**

We aimed to assess the accuracy of ChatGPT using GPT-3.5 and GPT-4 on the Peruvian National Licensing Medical Examination (Examen Nacional de Medicina [ENAM]). Additionally, we sought to identify factors associated with incorrect answers provided by ChatGPT.

**Methods:**

We used the ENAM 2022 data set, which consisted of 180 multiple-choice questions, to evaluate the performance of ChatGPT. Various prompts were used, and accuracy was evaluated. The performance of ChatGPT was compared to that of a sample of 1025 examinees. Factors such as question type, Peruvian-specific knowledge, discrimination, difficulty, quality of questions, and subject were analyzed to determine their influence on incorrect answers. Questions that received incorrect answers underwent a three-step process involving different prompts to explore the potential impact of adding roles and context on ChatGPT’s accuracy.

**Results:**

GPT-4 achieved an accuracy of 86% on the ENAM, followed by GPT-3.5 with 77%. The accuracy obtained by the 1025 examinees was 55%. There was a fair agreement (κ=0.38) between GPT-3.5 and GPT-4. Moderate-to-high-difficulty questions were associated with incorrect answers in the crude and adjusted model for GPT-3.5 (odds ratio [OR] 6.6, 95% CI 2.73-15.95) and GPT-4 (OR 33.23, 95% CI 4.3-257.12). After reinputting questions that received incorrect answers, GPT-3.5 went from 41 (100%) to 12 (29%) incorrect answers, and GPT-4 from 25 (100%) to 4 (16%).

**Conclusions:**

Our study found that ChatGPT (GPT-3.5 and GPT-4) can achieve expert-level performance on the ENAM, outperforming most of our examinees. We found fair agreement between both GPT-3.5 and GPT-4. Incorrect answers were associated with the difficulty of questions, which may resemble human performance. Furthermore, by reinputting questions that initially received incorrect answers with different prompts containing additional roles and context, ChatGPT achieved improved accuracy.

## Introduction

ChatGPT (OpenAI), a large language model (LLM) trained with over 175 billion parameters, has gained growing attention owing to its performance in different tasks, including mathematics, economics, and medicine [1]. During the first trimester of 2023, its performance in the United States Medical Licensing Examination (USMLE) has improved exponentially, from almost passing the USMLE Step 1 and Step 2 Clinical Knowledge with 40%-60% accuracy [2] to passing both with expert-level performance, achieving 80%-90% accuracy in a recent study with the latest ChatGPT version [3]. Even with recent communications from different organizations and authors on the potential of ChatGPT to improve accessibility to high-quality education [4], including medical education [5-7], more research is required on the performance of ChatGPT on the national licensing medical examination (NLME) from low-income countries.

In the Peruvian context, low-quality medical education is evidenced by high failure rates (42.8%) in the Peruvian NLME (Examen Nacional de Medicina [ENAM] in Spanish) [8]. This translates into lower-to-medium self-perceived competencies of Peruvian doctors in the treatment of mental health disorders [9], leadership and management skills [10], evidence-based medicine [11], and clinical practices [12]. Furthermore, the pupil-to-teacher ratio in tertiary education in Peru is 19:1, according to the World Bank, which is higher than the recommended 16:1. Although there are no studies on the training of clinical educators or medical teachers, we believe that the situation in Peru may be similar to that described in a study conducted on Israeli physicians, in which 65% reported that they did not receive any training in medical education [13]. In this context, ChatGPT may enhance Peruvian medical education, especially from students’ perspectives.

ENAM is a professional requirement for Peruvian medical doctors and international physicians who aspire to practice medicine within Peruvian borders. Since its introduction in 2003 by the Peruvian Society of Medical Schools, this examination has served as a key evaluation of doctors’ readiness to practice medicine in the country [14]. ENAM is a written assessment conducted in Spanish that follows a multiple-choice question format. The test, comprising 180 questions, is primarily based on clinical vignettes related to the most common diseases and health issues prevalent in Peru in clinical, surgical, and public health areas. For Peruvian doctors, this crucial exam is conducted at the end of their internship, culminating in their 7-year undergraduate medical training [14,15].

The passing score on the ENAM is 10.5 on a vigesimal scale (95/180). Over the years, the examination has gained even more significance owing to the regulatory measures that have made it a critical element in the selection process for Rural Service positions [16]. Additionally, ENAM scores heavily influenced the allocation of medical specialties, further underlining the role of the exam in shaping the professional paths of aspiring doctors in Peru. Therefore, passing the ENAM is not just about obtaining a license to practice medicine but also plays a considerable role in the professional trajectory of medical practitioners in the country.

Bearing this in mind, we hypothesized that if ChatGPT can pass the ENAM, it may be used as a medical tutor to enhance medical students’ experience. Thus, in this study, we aimed to assess the accuracy of ChatGPT (GPT-3.5 and GPT-4) on the ENAM and identify factors associated with incorrect answers provided by ChatGPT.

## Methods

### Data Set

Our primary data source was the 2022 ENAM question set obtained directly from the official website of the Peruvian Society of Medical Schools (ASPEFAM) [15]. The data set, comprising 180 multiple-choice questions, was subsequently uploaded to a Google Spreadsheet for evaluation. We refrained from translating the questions into English while maintaining their original Spanish language for authenticity and accuracy.

The 2022 data set was chosen for two main reasons: first, the ENAM blueprint ensures that each examination evaluates the same construct, thereby allowing a single year’s data to be representative; second, since ChatGPT’s training information only covers knowledge up to September 2021, the 2022 data set assures that the selected questions were not part of the model’s training data. Therefore, we assert that our data set selection strategy offers a degree of generalizability to the ENAM. The ENAM 2022 data set is available in Multimedia Appendix 1.

We carefully collected the exam questions and divided them into four parts: (1) stem, the main problem or story (for example, “A 75-year-old man...”); (2) lead-in, the question asked (for example, “What is the most probable diagnosis?”); (3) response options, the different answers provided for each question; and (4) the correct answer, as given by the exam creators [17].

### Procedures

Two ChatGPT versions were used, namely, GPT-3.5 and GPT-4. Our approach involved the development of three distinct prompts to guide the artificial intelligence (AI) response. To create these prompts, two authors (JAF-C and JG-A) engaged in discussions to ensure they accurately represented the cognitive processes an examinee would typically use when answering a multiple-choice question. After reaching a consensus, we designed a three-step prompt that, to the best of our understanding, mimics this thought process effectively.

The prompt was, “Analyze the following question, determine what is being assessed, and provide the correct answer/explanation.” With this prompt, we followed the same process as Kung et al [18], inputting questions in three formats:

1. Open-ended prompt: We removed response options, thus providing only the stem and lead-in with the prompt.
2. Multiple-choice question with no justification: We provided the whole question with a stem, lead-in, and response options. In the prompt, we asked only to provide the correct answer with no further explanation.
3. Multiple-choice question with justification: We provided the whole question with stem, lead-in, and response options. In the prompt, we asked for a lengthy explanation.

Five of us (four medical students and one medical doctor) entered the questions into ChatGPT. Students received training on how to use ChatGPT through a prerecorded video, and their proficiency was assessed to ensure consistency in the application of prompts. A new chat session was initiated for each question to eliminate any potential memory retention bias. In situations where ChatGPT initially failed to deliver a clear response, we reattempted the question up to three times. The responses were then transferred to a structured Google Spreadsheet for further examination. The first (GPT-3.5) data extraction process was conducted between March 15 and 20, 2023, and the second (GPT-4) was conducted on May 5, 2023.

On May 20, 2023, we conducted a second run, which incorporated three prompts following incorrect answers in GPT-3.5 and GPT-4. After providing the question and lead-in without instructions, if an incorrect answer was provided, we asked, “Are you sure? Pretend to be a junior doctor with expertise in clinical practice and exam solving and retry.” If an incorrect answer was provided, the following final prompt was provided: “Are you sure? Re-assess the question and pretend to be a Peruvian junior doctor with expertise in clinical practice and exam solving and retry.”

Additionally, we obtained the results of 1025 examinees who took the ENAM as a progress test in a national preparation course. The examinees comprised final-year medical students and medical doctors preparing to undertake the ENAM in 2023. Using this data set, we analyzed questions using classical test theory to calculate the difficulty and discrimination index using the psychometrics package in RStudio (version 4.2.1, RStudio, PBC). The difficulty index was calculated as a quantitative assessment of the proportion of examinees answering each question correctly, estimating the individual question’s difficulty level. The discrimination index refers to the question’s capacity to differentiate between high and low performers on the overall test [19]. These two metrics were used to assess the validity of an assessment and to distinguish between examinees, thus enabling us to evaluate the performance of ChatGPT more accurately.

### Variables

The outcome was the performance of ChatGPT (GPT-3.5 and GPT-4) on the ENAM measured as correct or incorrect answers. We classified answers as correct if the answer provided by both versions matched the official answers provided by ASPEFAM.

Independent variables were as follows: (1) type of objective, which was categorized as recall, whenever a question only required factual knowledge, or application, whenever a question required application of knowledge through clinical, therapeutic, communication, or professional decision-making; (2) Peruvian-specific knowledge (ie, if the question required knowledge specific to Peru, such as documentation or specific guidelines used in the country); (3) discrimination index; (4) difficulty index; (5) quality of questions; and (6) subject, which was categorized into basic sciences, internal medicine, surgery, obstetrics and gynecology, pediatrics, emergency medicine and critical care, and public health by two physicians with experience in assessing and preparing candidates for the ENAM. Both the discrimination and difficulty indices were calculated using classic test theory for the sample of 1025 examinees. For the discrimination index, we considered the question to provide good discrimination if the index was ≥0.25. For difficulty, questions were classified as hard (<0.30), moderate (0.30-0.70), or easy (>0.70). The quality of questions was measured by JAF-C and JG-A using a 5-point Likert scale with the question, “What is the quality of this question?”. Using this approach, we estimated the overall quality of the questions including the stem, lead-in, and response options using a tool based on the National Board of Medical Examinees’ item writing flaws [17].

### Statistical Analysis

We downloaded the data as Microsoft Excel files and exported the data to RStudio for analysis.

For descriptive analyses, we used absolute and relative frequencies for categorical variables and measures of central tendency and dispersion for numerical variables.

To compare the agreement between GPT-3.5 and GPT-4, we used Cohen κ. To evaluate factors associated with incorrect answers from GPT-3.5 and GPT-4, we used a logistic regression model to calculate the odds ratio (OR) and 95% CI.

We used the variance inflation factor (VIF) and Hosmer-Lemeshow test for goodness of fit to assess multicollinearity among predictors. All variables of interest were entered into the multivariable model, and this process was conducted for GPT-3.5 and GPT-4. The predictive accuracy of each version of ChatGPT was assessed using the receiver operating characteristic (ROC), from which we calculated the area under the curve (AUC). The data set and the RStudio script are available in Multimedia Appendices 2 and 3, respectively.

### Ethical Considerations

This study adhered to the Helsinki Declaration. No humans were involved during the study. Therefore, evaluation by the ethics committee was not considered necessary.

## Results

### Overall Performance

**Figure 1:**
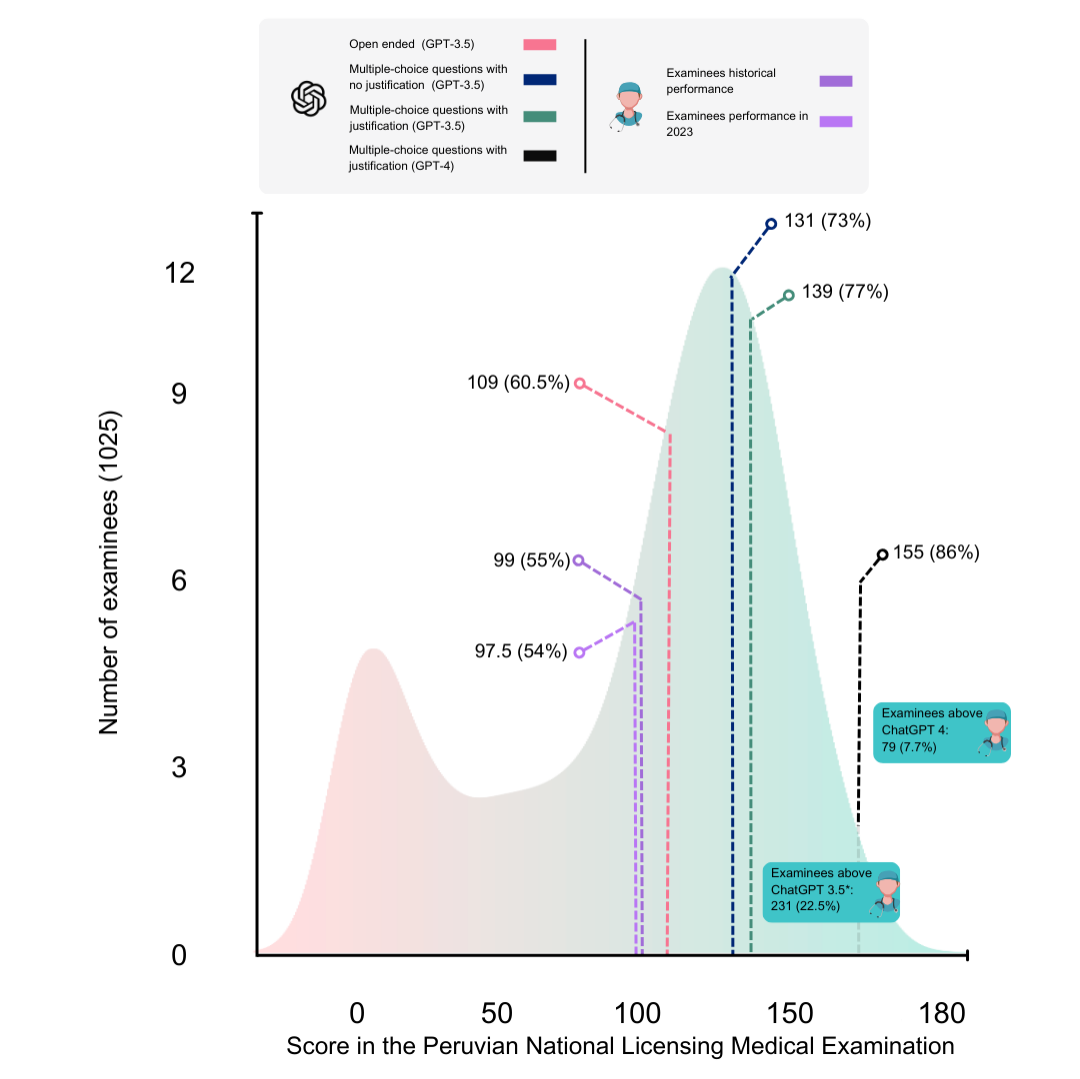
Performance of ChatGPT compared with 1025 examinees' scores.

The performance of GPT-4 was 86% (155/180). GPT-3.5 scored 77% (139/180), 73% (133/180), and 60.5% (109/180) for multiple-choice questions with justification, multiple-choice questions with no justification, and open-ended prompts, respectively. The historical performance of the examinees was 54% (97.5/180), and the examinees’ performance in our data set was 55% (99/180). Additionally, we calculated that 7.7% (79/1025) and 22.53% (231/1025) of examinees scored better than GPT-3.5 and GPT-4, respectively, as shown in Figure 1.

### Comparison of GPT-3.5 and GPT-4

**Figure 2:**
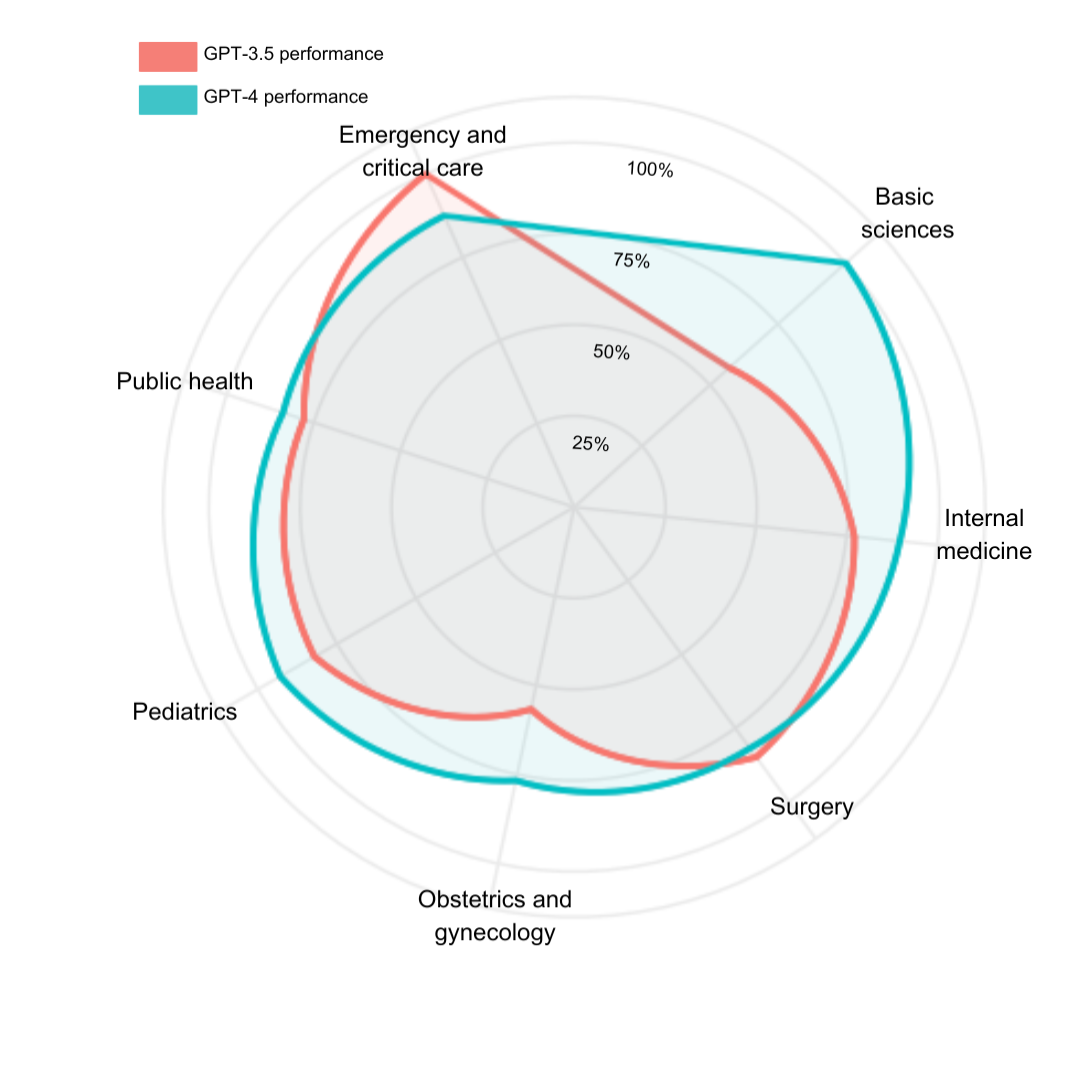
Performance of GPT-3.5 and GPT-4 in specific medical areas.

As shown in Figure 2, GPT-4 outperformed GPT-3.5 in almost all medical areas except surgery (GPT-4, 81.8%; GPT-3.5, 84.8%) and emergency medicine (GPT-4, 87.5%; GPT-3.5, 100%); however, these differences were not significant. When conducting a subanalysis for each subcategory, we found that GPT-4 outperformed GPT-3.5 in all categories except for medium-quality questions, as shown in Table 1.

**Table 1:** Correct answers provided by GPT-3.5 and GPT-4.

Characteristics			ChatGPT-3.5^a^ Correct answers, n (%)		ChatGPT-4 correct answers, n (%)		Cohen κ coefficient
Overall (N=180)			139 (77.2)		155 (86.1)		0.38
**Required knowledge from Peruvian context**							
	Yes (n=13)	11 (76.6)		10 (76.9)		0.76	
	No (n=167)	128 (84.6)		145 (86.8)		0.35	
**Area**							
	Basic sciences (n=7)	4 (57.1)		7 (100)		57.1%^b^	
	Internal medicine (n=48)	37 (77.1)		43 (89.6)		0.27	
	Surgery (n=33)	28 (84.8)		27 (81.8)		0.46	
	Obstetrics and gynecology (n=30)	17 (56.7)		23 (76.7)		0.57	
	Pediatrics (n=28)	23 (82.1)		26 (92.9)		0.02	
	Public health (n=18)	14 (77.8)		15 (83.3)		0.47	
	Emergency and critical care (n=16)	16 (100)		14 (87.5)		87.5%^b^	
**Quality of questions**							
	Low quality (n=29)	27 (82.8)		26 (89.7)		0.35	
	Medium quality (n=38)	25 (81.6)		30 (78.9)		0.42	
	High quality (n=99)	76 (74.7)		85 (85.9)		0.38	
	Very high quality (n=14)	11 (71.4)		14 (100)		78.6%^b^	
**Bloom Taxonomy**							
	Recall (n=50)	38 (76)		40 (80)		0.65	
	Application (n=130)	101 (77.7)		115 (88.5)		0.25	
**Discrimination**							
	Good discrimination index (≥0.25; n=123)	93 (75.6)		105 (85.4)		0.34	
	Bad discrimination index (<0.25; n=57)	46 (80.7)		50 (87.7)		0.48	
**Difficulty**							
	High difficulty index (<0.3; n=2)	0 (0)		0 (0)		100%^b^	
	Moderate difficulty index (0.3-0.7; n=86)	55 (64)		64 (74.4)		0.33	
	Low difficulty index (>0.7; n=92)	84 (91.3)		91 (98.9)		90%^b^	

^a^Prompts were formatted as multiple-choice questions with justification.

^b^Proportion of agreement between raters. This was calculated when Cohen κ calculation was not feasible.

**Figure 3:**
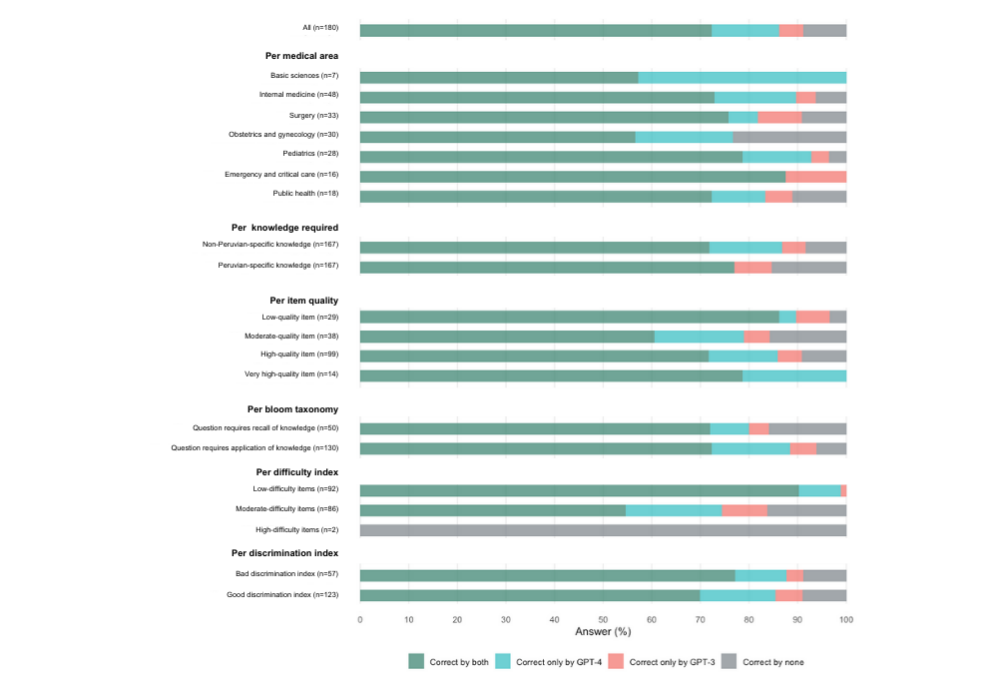
GPT-3.5 and GPT-4 response agreement.

We used Cohen κ to assess the agreement between GPT-3.5 and GPT-4; the overall agreement was κ=0.38 (Table 1). The agreement was higher for questions that required Peruvian knowledge (κ=0.76), questions that assessed recall of knowledge (κ=0.65), and questions from obstetrics and gynecology (κ=0.57). When calculating Cohen κ was not feasible, we calculated the proportion of agreement between raters, which was highest for high-difficulty questions (100%), low-difficulty questions (90%), and questions from emergency and critical care (87.5%). A more in-depth analysis is portrayed in Figure 3.

### Factors Associated With ChatGPT Incorrect Answers

When analyzing the odds for incorrect answers on GPT-3.5 and GPT-4, we found that high- and moderate-difficulty questions presented higher odds for incorrect answers in the adjusted model both for GPT-3.5 (OR 6.6, 95% CI 2.73-15.95) and GPT-4 (OR 33.23, 95% CI 4.3-257.12), and low-quality questions were associated with correct answers in the GPT-3.5 adjusted model (OR 0.14, 95% CI 0.02-0.87), as shown in Table 2. Furthermore, the GPT-3.5 and GPT-4 adjusted models had AUCs of 0.782 and 0.851, respectively. None of the variables included had a VIF>5.

**Table 2:** Factors associated with incorrect answers given by GPT-3.5 and GPT-4^a^.

			Incorrect, n (%)		Crude OR^b^ (95% CI)		Adjusted^c^ OR (95% CI)	Incorrect, n (%)		Crude OR (95% CI)		Adjusted OR (95% CI)
**Peru-specific knowledge required**												
	No	39 (23.4)		Ref^d^		N/A^e^		22 (13.2)	Ref		N/A	
	Yes	2 (15.4)		0.6 (0.13-2.81)		0.65 (0.11-3.81)		3 (23.1)	1.98 (0.5-7.75)		2.05 (0.36-11.61)	
**Area**												
	Clinical areas^f^	16 (21.1)		Ref		N/A		7 (10.5)	Ref		N/A	
	Surgical areas^g^	18 (28.6)		1.5 0(0.69-3.29)		1.36 (0.56-3.29)		13 (20.6)	2.56 (0.95-6.88)		2.32 (0.75-7.15)	
	Longitudinal areas^h^	7 (17.1)		0.77 (0.29-2.06)		0.77 (0.24-2.48)		5 (12.2)	1.37 (0.41-4.62)		0.89 (0.2-4.01)	
**Quality of questions**												
	High quality	23 (20.2)		Ref		N/A		14 (14.1)	Ref		N/A	
	Low quality	2 (20.7)		0.24 (0.05-1.11)		0.14 (0.02-0.87)*		3 (10.3)	0.70 (0.23-2.12)		0.28 (0.04-1.89)	
	Medium quality	13 (28.9)		1.72 (0.76-3.89)		1.08 (0.38-3.07)		8 (21.1)	1.62 (0.62-4.24)		0.83 (0.21-3.19)	
	Very high quality	3 (28.6)		0.90 (0.23-3.51)		1.28 (0.27-5.99)		0 (0)	—^i^		—	
**Bloom Taxonomy**												
	Application	29 (22.3)		Ref		N/A		15 (11.5)	Ref		N/A	
	Recall	12 (24)		1.1 (0.51-2.37)		1.76 (0.53-5.82)		10 (20)	1.92 (0.8-4.61)		2.05 (0.36-11.61)	
**Discrimination**												
	Good discrimination index (≥0.25)	30 (24.4)		Ref		N/A		18 (14.6)	Ref		N/A	
	Bad discrimination index (<0.25)	11 (19.3)		0.74 (0.34-1.61)		0.92 (0.38-2.24)		7 (12.3)	0.82 (0.32-2.08)		1.07 (0.34-3.36)	
**Difficulty**												
	Low difficulty index (>0.7)	8 (8.7)		Ref		N/A		1 (1.1)	Ref		N/A	
	High and moderate difficulty index (≤0.7)	33 (37.5)		6.3 (2.71-14.65)*		6.6 (2.73-15.95)*		24 (27.3)	34.12 (4.5-258.95)*		33.23 (4.3-257.12)*	

^a^The area under the curve was 0.782 for GPT-3.5 and 0.851 for GPT-4. The variance inflation factor was <5 for all variables.

^b^OR: odds ratio.

^c^Model adjusted by Peru-specific knowledge requirement, area, quality of questions, bloom taxonomy, discrimination, and difficulty.

^d^Ref: reference category.

^e^N/A: not applicable.

^f^Clinical areas include internal medicine and pediatrics.

^g^Surgical areas include obstetrics and gynecology and surgery.

^h^Longitudinal areas include public health, basic sciences, and emergency and critical care.

^i^Not available.

*P<.05.

### Reinput of Prompts for Incorrect Answers

**Figure 4:**
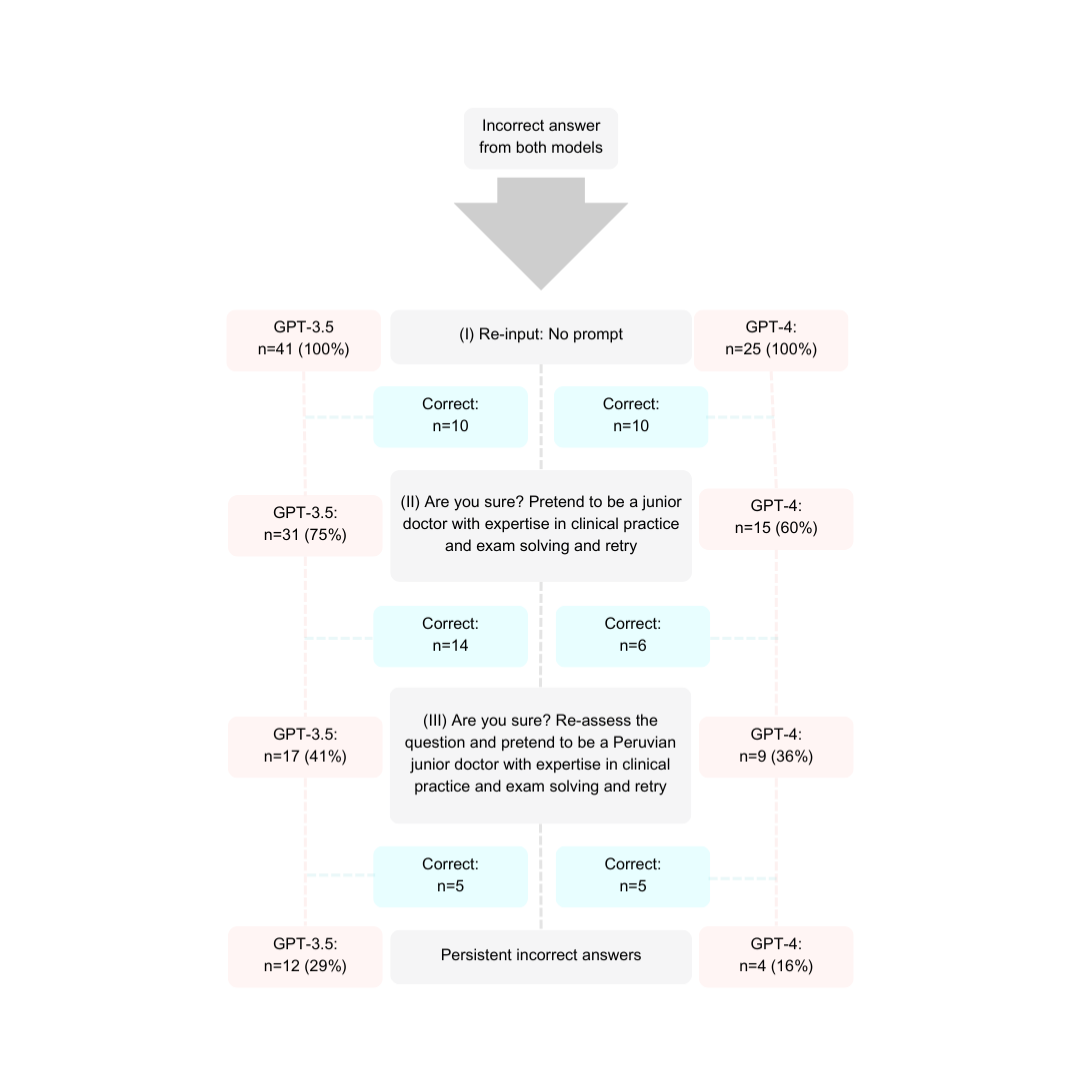
Flowchart of the reinput process for incorrect answers provided by GPT-3.5 and GPT-4.

Finally, we reinput prompts for incorrect answers following a three-step process, as shown in Figure 4. After reinputting prompts, GPT-3.5 provided 12 (29%) persistent incorrect answers, and GPT-4 provided 4 (16%), thus exhibiting improved scores when modeled through different prompts.

## Discussion

### Principal Findings

Here we showed that ChatGPT (GPT-3.5 and GPT-4) can pass the ENAM with expert-level performance. Furthermore, GPT-4 surpassed almost 90% of examinees in our data set with an accuracy of 86.1%, and GPT-3.5 surpassed 80% of examinees with an accuracy of 77.2%. These results are in concordance with the findings of Nori et al [3], who reported an accuracy of 84.75% and 48.12% for GPT-4 and GPT-3.5, respectively, in the USMLE Step 2 Clinical Knowledge. Another study on the Neurosurgery Oral Board Preparation Question Bank showed that GPT-4 performed with an accuracy of 82.6%, while GPT-3.5 achieved an accuracy of 62.4% [20]. However, in our study, GPT-3.5 performed better on the NLME compared to previous studies where it failed examinations, including the USMLE and Spanish, Japanese, and Chinese NLMEs [2,21-23]. This can be explained by our use of a prompt that resembles the “chain-of-thought prompting approach,” in which ChatGPT decomposes multistep problems into smaller and manageable steps to enhance accuracy [24]. However, more studies are needed to understand whether this prompt structure improves performance in health care–related tasks.

When analyzing differences between the two versions, GPT-4 outperformed GPT-3.5 in almost all areas; however, we observed fair agreement between versions. The agreement was higher for high-difficulty questions, for which both versions failed all questions, and low-difficulty questions, for which both versions answered all questions correctly. These results suggest that the improvement in performance from GPT-3.5 to GPT-4 is due to enhanced reasoning rather than randomness [1].

Although previous studies reported the likelihood of lower accuracy in GPT-3.5 for higher-order problem-solving [20], we found that when adjusting for all variables, moderate-to-high difficulty questions were associated with incorrect answers for both GPT-3.5 and GPT-4 and that low-quality questions were associated with correct answers for only GPT-3.5. Notably, our findings differ from those of another study that did not find a correlation between question difficulty and accuracy using GPT-3.5 [25]; however, in that study, difficulty was measured through perception rather than through classic test theory. Lastly, we showed that when reinputting questions, ChatGPT provided new and more accurate responses and that role-play and context-setting in prompts effectively improved performance, reducing GTP-3.5’s incorrect answers from 41 to 12 and GTP-4’s incorrect answers from 25 to 4. Our findings resemble those of a previous study that showed that novel explanations provided when reinputting questions improved performance from 8.61% to 9.79% [25].

### Strengths and Limitations

To our knowledge, this is the first study to assess the agreement between GPT-3.5 and GPT-4 in the context of medical education and to examine factors linked to incorrect answers. We demonstrated that reformulating incorrect answers by varying prompts and changing roles and contexts improved the accuracy of ChatGPT.

However, certain limitations of this study should be considered when interpreting our results. First, our study was confined to the Peruvian medical education system and involved a relatively limited number of questions. Therefore, the results may not be generalizable to other educational settings or a wider range of questions. We recommend future research with larger sample sizes, more diverse examinations, broader question sets, and different factors to identify reasons for wrong answers, such as the date of the questions.

Second, while GPT-4 exhibited expert-level performance on the ENAM, this finding must be cautiously interpreted. The competencies required by a medical professional, as defined by frameworks such as CanMEDS or the Accreditation Council for Graduate Medical Education core competencies, extend beyond the confines of a licensing examination. These examinations assess knowledge and its application under controlled conditions, which may differ substantially from real-world clinical scenarios. Furthermore, more valid assessment tools, such as entrustable professional activities, represent the gold standard in medical education. Consequently, despite GPT-4’s promising performance, it is premature to suggest that it could replace human doctors. We encourage additional research to assess the potential use of ChatGPT in different roles or as a supportive tool for medical practitioners.

Finally, our study did not evaluate the use of “mega-prompts”—large, intricate prompts detailing specific roles, contexts, and tasks, which might elicit more sophisticated and targeted responses—or other novel methods, such as chain-of-thought prompts [24] or three-of-thoughts [26]. Therefore, our findings may not fully encompass the range and depth of responses that GPT-3.5 and GPT-4 can achieve. We recommend that future studies explore the effects of different prompts on the performance of ChatGPT in medical education.

### Implications

This study has several implications for both medical education and research on ChatGPT and AI. First, we demonstrated that ChatGPT can pass the ENAM with expert-level performance, surpassing 9 out of 10 examinees. Although our sample does not represent the real score in the ENAM, a previous study [9] found that high ENAM scores from examinees from 2009 to 2019 ranged between 16.58-17.63, which is on par with GPT-4’s score of 17.2. Using a variety of LLMs, we can begin to tailor assessments for different students’ needs, as each LLM (InstructGPT, GPT-3.5, GPT-4, or others) may be representative of a cluster of subjects or performance levels from novice to expert. Thus, assessments may be inputted into LLMs, and an ease-rapid-valid evaluation of the level of the assessment may be estimated using the percentage of correct answers obtained by the selected LLM.

Second, we found that incorrect answers provided by ChatGPT using GPT-3.5 and GPT-4 were associated with question difficulty, which opens further research directions to identify reasons for why ChatGPT fails some questions and inform new directions to understand the behavior of LLMs. Also, to our knowledge, this study is the first to apply psychometrics to ChatGPT, and further studies could explore different theories, such as cognitive diagnostic modeling or other diagnostic classification models with larger data sets, searching for a more in-depth understanding of the reasoning process of ChatGPT.

Third, by reinputting incorrectly answered questions and adjusting prompts with more complexity (ie, adding roles and context), we found that ChatGPT may perform better. This requires further research on prompt engineering in medical education with tailored prompts for specific tasks, such as the development of assessment tools, curriculum development, communication with patients, or tutoring students. Additionally, tailored LLMs trained with specific and curated medical knowledge are needed for these different applications.

Finally, despite the outstanding performance of ChatGPT in the ENAM, as previously stated by Thirunavukarasu [27], practicing medicine requires more than just responding correctly to a set of multiple-choice questions. Thus, being a doctor is a complex and never-ending process that requires us to wear several hats as medical experts, communicators, collaborators, academics, and several other roles. Consequently, we recommend that future research be aligned with medical competencies and roles; this will allow us to guide research on ChatGPT and LLMs to answer more specific questions that may aid us in spending time on more meaningful tasks.

### Conclusions

Our study found that ChatGPT (GPT-3.5 and GPT-4) can achieve expert-level performance on the ENAM, outperforming most of our examinees. We found fair agreement between both versions. There was an association between high-to-moderate-difficulty questions and wrong answers in both versions of ChatGPT. Furthermore, we observed enhanced performance by reinputting new prompts for incorrectly answered questions and adding roles and context for ChatGPT. Despite the outstanding performance of ChatGPT, we note that being a doctor goes beyond passing a licensing examination.

## Appendix

Multimedia Appendix 1 Examen Nacional de Medicina 2022 data set.

Multimedia Appendix 2 Data set.

Multimedia Appendix 3 RStudio script.

## Abbreviations

AI: artificial intelligence
AUC: area under the curve
ENAM: Examen Nacional de Medicina
LLM: large language model
NLME: national licensing medical examination
OR: odds ratio
ROC: receiver operating characteristic
USMLE: United States Medical Licensing Examination
VIF: variance inflation factor
